# Gendered health consequences of unemployment in Norway 2000–2017: a register-based study of hospital admissions, health-related benefit utilisation, and mortality

**DOI:** 10.1186/s12889-022-14899-8

**Published:** 2022-12-28

**Authors:** Kristian Heggebø

**Affiliations:** 1 NOVA, OsloMet, Oslo Metropolitan University, Oslo, Norway; 2 CHAIN, NTNU, Norwegian University of Science and Technology, Trondheim, Norway

**Keywords:** Health effects, Unemployment, Labour market, Gender differences, Registry data, Time trends, Nordic welfare state

## Abstract

**Background:**

The existing literature indicates that unemployment leads to deteriorated mental and somatic health, poorer self-assessed health, and higher mortality. However, it is not clear whether and to what extent the health consequences of unemployment differ between men and women. According to social role theory, women can alternate between several roles (mother, wife, friend, etc.) that make it easier to deal with unemployment, whereas the worker role is more important for men, and unemployment could therefore be more harmful to them. Thus, gender differences in the health consequences of unemployment should decrease as society grows more gender equal. Accordingly, this study examines changes over time in the gendered health consequences of unemployment in Norway.

**Methods:**

Linked Norwegian administrative register data, covering the period from 2000 to 2017, were analysed by means of linear probability models and logistic regression. Four health outcomes were investigated: hospitalisation, receiving sick pay, disability benefit utilisation, and the likelihood of mortality. Two statistical models were estimated: adjusted for (1) age, and (2) additional sociodemographic covariates. All analyses were run split by gender. Three different unemployment cohorts (2000, 2006, and 2011) that experienced similar economic conditions were followed longitudinally until 2017.

**Results:**

The empirical findings show, first, that hospital admission is somewhat more common among unemployed males than among unemployed females. Second, receiving sick pay is much more common post-unemployment for men than for women. Third, excess mortality is higher among unemployed males than among unemployed females. Fourth, there is no gender component in disability benefit utilisation. There is a remarkable pattern of similarity when comparing the results for the three different unemployment cohorts (2000; 2006; 2011). Thus, the gendered health consequences of unemployment have hardly changed since the turn of the century.

**Conclusion:**

This paper demonstrates that the health consequences of unemployment are serious, gendered, and enduring in Norway.

**Supplementary Information:**

The online version contains supplementary material available at 10.1186/s12889-022-14899-8.

## Background

It is well documented that unemployment is associated with health deterioration [1]. Mental health is affected by unemployment [2–4], leading to, for example, depression and anxiety [5]. Unemployed people report poorer self-assessed health [6, 7] and utilize healthcare services more often [8–10]. Excess mortality also tends to be high among the unemployed [11, 12]. Furthermore, cross-national comparative studies have revealed that unemployment is harmful for health in all countries covered [13, 14].

Financial strain appears to play a role in explaining why mental health is affected by unemployment [15, 16], whereas elevated levels of inflammatory markers (e.g., C-reactive protein and interleukin 6) represent one path from unemployment to somatic health conditions [17–19]. Health-related social mobility – that is, the impact of poor health status on, for example, educational attainment, occupational careers, and job loss likelihood – is also of importance for the strength of the unemployment-health association [20, 21].

A handful of previous studies have found no evidence of a negative causal health effect of unemployment within a counterfactual framework [22–24]. However, there is broad agreement overall in the existing literature that unemployment is harmful for various aspects of health [1–19]. Whether the health consequences of unemployment differ between males and females is more disputed, though. Some studies have found that men are more prone to health consequences than women post-unemployment [13, 25–28], whereas other studies have found that women are equally or more affected by unemployment [9, 29–33].

Inconsistencies in the existing literature can probably be explained partly by differing data materials, health outcomes, and length of follow-up. The ‘gender health paradox’ could be one potential explanation. Females report more health problems and utilise healthcare services more often, whereas males die earlier, on average[34, 35]. This paradox may, to some extent, explain why existing studies disagree on how gendered the health consequences of unemployment really are. For example, two studies from Sweden published in 2011–12 showed diverging findings [27, 31]. On the one hand, a register-based study reported that the effects of unemployment on all-cause mortality are more pronounced among men than women [27]. A survey-based study, on the other hand, indicated more self-reported mental health problems among unemployed females compared to their male counterparts [31]. Comparing results across differing health outcomes could therefore reveal important insights.

The presence or absence of strong cultural expectations for the man to be the main financial provider for his family (i.e., the male breadwinner model) might matter as well [36, 37]. According to social role theory, women have multiple roles to alternate between (i.e., mother, wife, friend, worker), whereas the worker role tends to be more crucial for men [38, 39]. Since women can alternate between, and gain recognition from, various social roles, it may be easier for women to deal with the experience of unemployment. Men’s social identity, by comparison, is more tightly connected to work and employment, and job loss could therefore prove to be more harmful. However, gender differences in the importance of the worker role have probably become smaller over time, as, for example, women have increasingly entered (previously) male-dominated occupations, female rates of part-time work have decreased, men take more responsibility for childrearing and housework, et cetera. Accordingly, gender differences in the health consequences of unemployment should decrease over time as society grows gradually more gender equal.

Social role theory also predicts that health consequences will be particularly gendered (i.e., men experience graver consequences) in countries and regions where the male breadwinner model prevails. Conversely, we should observe rather small gender differences in countries and regions where gender norms are comparatively egalitarian. Two recent papers [40, 41] analysing the German Socio-Economic Panel found some support for this theoretical model, showing that males are hurt more by unemployment than females, but only in the former West Germany. The differences between men and women were negligible for respondents who grew up in East Germany, where there is a longer tradition of female labour force participation and gender egalitarianism due to its socialist past.

The current study attempts to move these discussions forward by illuminating the gendered health consequences of unemployment after the turn of the century in one of the most gender-egalitarian countries in the world: Norway [42, 43]. Numerous linked administrative register data sources were analysed in order to answer the following overarching research question: How gendered are the health consequences of unemployment in Norway from 2000 to 2017?

Four health outcomes were examined longitudinally, which correspond with the aetiology of mental and somatic health conditions that may arise due to unemployment and associated stress and worries. The first outcome is hospital admissions due to mental and behavioural disorders; diseases of the nervous, circulatory, and respiratory systems; and injuries, poisoning, and other external causes. Second, we looked at receiving sick pay (i.e., a temporary health-related benefit). Third, we analysed disability benefit utilisation (i.e., a permanent health-related benefit). Fourth and finally, we examined the 10-year mortality likelihood. We analysed, by means of linear probability models and logistic regression, health trajectories over time among people that received unemployment benefits in three different exposure years: 2000, 2006, and 2011. The three exposure years were chosen because the economic conditions were very similar, which should ease cross-cohort comparisons. Based on the literature review and theoretical reflections above, two main hypotheses can be derived:

- H1: Gender differences in the health consequences of unemployment are greater for mortality than for the other health outcomes, in accordance with the ‘gender health paradox’.
- H2: Gender differences in the health consequences of unemployment have decreased over time, as the Norwegian society has gradually become more gender equal.

The current study aims to add to the existing literature in three domains. First, by analysing four register-based outcomes (hospitalisation, receiving sick pay, disability benefit utilisation, and mortality), which together will hopefully paint a comprehensive picture of the gendered health consequences of unemployment in Norway. Second, by following unemployed cohorts longitudinally, covering an 18-year period, we can examine the medium-to-long-term consequences of unemployment, which represents a gap in previous research, according to Norström et al. [33]. Third, we can examine potential time trends by reporting empirical findings for three unemployed cohorts that experienced unemployment during different times (2000; 2006; 2011) yet with similar economic conditions.

## Methods

### Register data and sample inclusion criteria

The current study uses numerous Norwegian administrative register data sources, such as the Norwegian Patient Registry (NPR) and FD-Trygd (‘longitudinal social security data’), which cover all registered inhabitants in Norway. The population-wide register data are linked by Statistics Norway via project-specific deidentified serial numbers derived from unique personal identification numbers. It was not necessary to obtain informed consent, since the data are in anonymised and deidentified format, in accordance with Norwegian privacy legislation. The current research was approved by the Norwegian Agency for Shared Services in Education and Research and by the Regional Committees for Medical and Health Research Ethics.

The observational period is 2000 to 2017, yet some variables are not available for the entire 18-year period. For example, information on hospitalisations from the NPR are only available from 2008 to 2017. Those who were in the labour market, either employed or unemployed as defined below, between the ages of 25 and 55 in 2000/2006/2011 were included in three analytical samples. The maximum age is 72 (55 + 17 follow-up years, for the 2000 cohort). Persons who had emigrated were excluded. Persons who had died were excluded from the analyses of hospitalisation and health-related benefit utilisation. All three analytical samples were followed year-by-year longitudinally in terms of health outcomes until 2017 (e.g., 11 follow-up years for the 2006 cohort).

### Operationalisation

Four health outcomes were examined. First, in- and out-patient hospitalisation was analysed for at least one of the following five International Classification of Diseases (ICD) code groups (yes = 1, no = 0): mental and behavioural disorders (F00-F99); diseases of the nervous system (G00-G99); diseases of the circulatory system (I00-I99); diseases of the respiratory system (J00-J99); and injury, poisoning, and other external causes (S00-T98). Second, we examined physician-certified sick pay receipt (more than zero Norwegian krone (NOK) received = 1, else = 0). Short-term sickness absences of 16 days or less were not included. Third, we analysed disability benefit utilisation (more than zero NOK received = 1, else = 0). Fourth and finally, we looked at the 10-year (and 6-year) mortality likelihood (yes = 1, no = 0).

The unemployed cohorts were identified via unemployment benefit receipt during a calendar year (more than zero NOK received = 1, else = 0). Throughout, the control group consisted of individuals who were employed and earned more than 3.5 times the base amount (BA) in work income in 2000/2006/2011. The BA is a sum of money set by the Norwegian Parliament each year and used, for example, to calculate benefit levels. An amount of 3.5 BA corresponds roughly to what a full-time worker in the low-income bracket earns yearly.

Model 1 was adjusted for age in years and age squared. Model 2 controlled for age, age squared, marital status (married = 1, else = 0), immigrant background (born abroad = 1, else = 0), and two educational level dummies: high education (International Standard Classification of Education (ISCED)-levels 6–8 = 1, else = 0), and medium education (ISCED-levels 4–5 = 1; else = 0). The category of low education (ISCED-levels 0–3) was omitted. Other socioeconomic indicators (e.g., occupational class or income) were not included to circumvent interpretation problems stemming from multi-collinearity issues [44].

### Statistical methods and analysis design

The linked register data were analysed by means of linear probability models, that is, ordinary least squares (OLS) regression of a dichotomous outcome. Odds ratios (OR) derived from logistic regression were reported for the analysis of mortality to ease comparison with previous research. However, linear probability models were also performed, since odds ratios are difficult to compare across different samples and model specifications [45, 46]. The findings from both the age-adjusted models (model 1) and the models with additional adjustment for sociodemographic covariates (model 2) are presented throughout. All models were performed split by gender and displayed as figures with 95% confidence intervals to analyse whether the gender differences are statistically significant. The statistical models were estimated for each calendar year, and the coefficient of interest, the unemployed dummy, is reported for men and women separately. Thus, for the 2000 unemployed cohort, a total of 17 coefficients are reported (i.e., one for each year during the period 2001–2017), if data are available for the entire period.

As mentioned above, it is conceivable that the gender differences in health consequences of unemployment are decreasing over time as Norwegian society becomes increasingly gender equal. The unemployment-health association may also change with the passage of time due to, for example, demographic developments, increasing economic inequalities, and other underlying time trends. To examine whether the gendered health consequences of unemployment have changed noticeably, we analyse health trajectories among unemployment benefit recipients in 2000, 2006, and 2011. These three years were chosen because the economic conditions, as indicated by the unemployment rate, were very similar. According to official figures on registered unemployment, the yearly average unemployment rate was 2.7% in 2000, 2.6% in 2006, and 2.7% in 2011 [47]. Figures from the Norwegian Labour Force Survey paint a similar picture: 3.4% of the workforce was unemployed in all three years [48].

Previous research has revealed that the unemployment-health association is sensitive to prevailing economic conditions [49, 50]. We chose exposure years with as similar economic conditions as possible to minimise cross-cohort variation, for instance compositional differences in unobservable characteristics such as personality or work motivation. Thus, this comparative design tries to isolate the time trend as much as possible. However, uncertainties remain as it is not possible to test the underlying assumption, that is, that the three unemployed cohorts (2000; 2006; 2011) are similar, or at least comparable.

## Results

### Summary statistics

Table 1 shows that approximately 1.3 million employed people, men and women combined, were included in the analytical samples for all three exposure years (2000;2006;2011). The unemployed are married to a lesser extent, have high education less often, and are slightly younger on average than the employed. Immigrants are overrepresented (by more than double) among the unemployed in all three cohorts. As is evident from Table 2, there are few gender differences in sociodemographic characteristics, with two exceptions: female unemployed are more often married and less often have an immigrant background compared to male unemployed, in all three cohorts. The number of unemployed aged 25–55 in the three cohorts varies between 43,641 and 81,228 for males, and between 46,602 and 60,533 for females.

**Table 1:** Summary statistics for employed and unemployed cohorts (age 25–55) in 2000, 2006, and 2011

	2000 cohort		2006 cohort		2011 cohort	
	Employed	Unemployed	Employed	Unemployed	Employed	Unemployed
**Outcomes**						
Hospitalisation	13.00	15.75^a^	15.47	19.05^a^	16.51	19.37^a^
Sick pay	28.57	30.09^a^	28.21	31.51^a^	26.73	26.33^a^
Disability benefit	3.50	5.88^a^	2.05	3.96^a^	1.95	4.26^a^
Mortality	0.81	1.17^a^	0.67	0.96^a^	0.57	0.80^a^
**Covariates**						
Woman	40.63	44.58^a^	42.13	51.64^a^	43.93	39.74^a^
Age (in years)	40.15	37.23^a^	40.57	37.52^a^	40.86	38.00^a^
Married	54.49	38.75^a^	50.28	36.39^a^	48.39	36.64^a^
Immigrant	6.38	12.87^a^	8.36	18.24^a^	12.48	29.71^a^
Education						
*High*	35.22	16.19^a^	39.87	23.83^a^	44.29	22.48^a^
*Medium*	30.91	32.23^a^	34.59	33.28^a^	35.65	36.47^a^
*Low*	34.46	50.53^a^	24.65	40.02^a^	18.62	35.08^a^
N	1,284,679	135,785	1,306,814	90,243	1,355,186	134,785

Outcomes were measured after six years (i.e., in 2007/2012/2017), with one exception: hospitalisation for the 2000 cohort (2008 first observational year)

^a^statistically significant within-cohort difference (95% level)

**Table 2:** Summary statistics for 2000, 2006, and 2011 unemployment cohorts (age 25–55), split by gender

	2000 cohort		2006 cohort		2011 cohort	
	Males	Females	Males	Females	Males	Females
**Outcomes**						
Hospitalisation	16.35	15.00^a^	19.68	18.47^a^	19.03	19.88^a^
Sick pay	25.96	35.23^a^	25.37	37.25^a^	22.22	32.55^a^
Disability benefit	5.66	6.15^a^	3.74	4.17^a^	3.83	4.92^a^
Mortality	1.62	0.62^a^	1.40	0.56^a^	1.04	0.43^a^
**Covariates**						
Age (in years)	37.29	37.16^a^	37.55	37.48	38.13	37.81^a^
Married	32.40	46.64^a^	30.31	42.08^a^	34.18	40.36^a^
Immigrant	14.25	11.15^a^	20.54	16.09^a^	32.61	25.30^a^
Education						
*High*	14.88	17.81^a^	19.91	27.51^a^	17.06	30.70^a^
*Medium*	34.99	28.80^a^	33.59	32.99^a^	38.20	33.84^a^
*Low*	48.74	52.74^a^	43.13	37.10^a^	37.12	31.99^a^
N	75,252	60,533	43,641	46,602	81,228	53,557

Outcomes were measured after six years (i.e., in 2007/2012/2017), with one exception: hospitalisation for the 2000 cohort (2008 first observational year)

^a^statistically significant within-cohort gender difference (95% level)

### Regression results

**Fig. 1:**
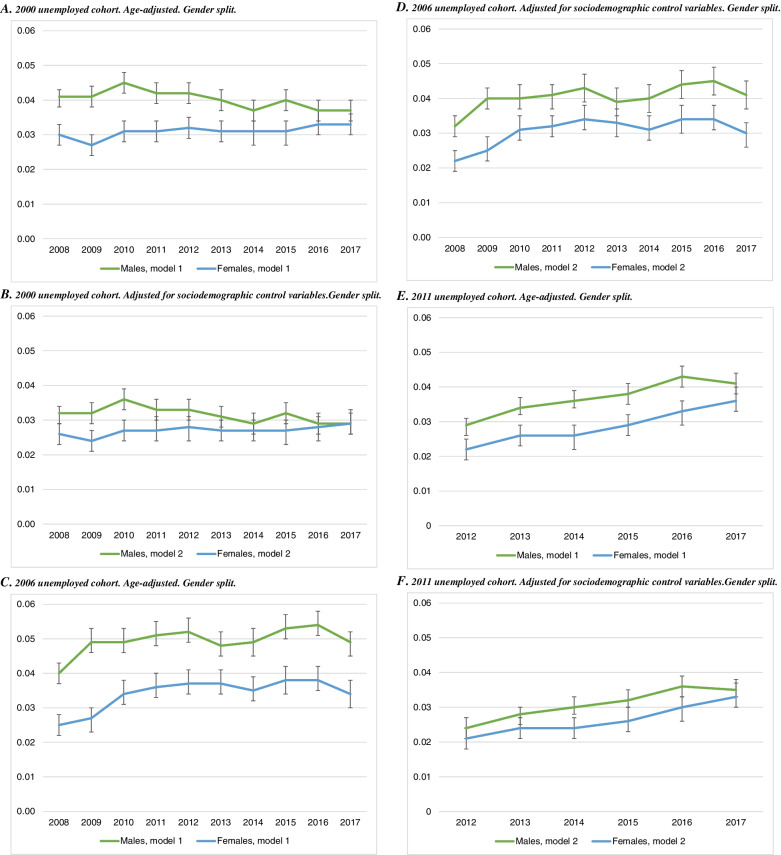
Linear probability models of hospitalisation in 2008–2017, by unemployment

Figure 1 presents the empirical findings for hospitalisation. Starting with the 2000 unemployed cohort (panel A), males have a roughly 4 percentage points higher hospitalisation likelihood during 2008–2017. The corresponding difference is lower, at about 3 percentage points, for females. The gender differences are smaller after adjustment for sociodemographic covariates (Fig. 1, panel B), and especially during 2013–2017 when the confidence intervals overlap. The coefficients are somewhat larger for both males and females (at roughly 5 and 3.5 percentage points, respectively) in the 2006 cohort (panels C and D). In the most recent unemployed cohort (panels E and F), the coefficients are initially small, but increase to roughly 4 (males) and 3,5 (females) percentage points in 2016–2017. For all three cohorts, the gender differences are attenuated after control for sociodemographic covariates in model 2.

**Fig. 2:**
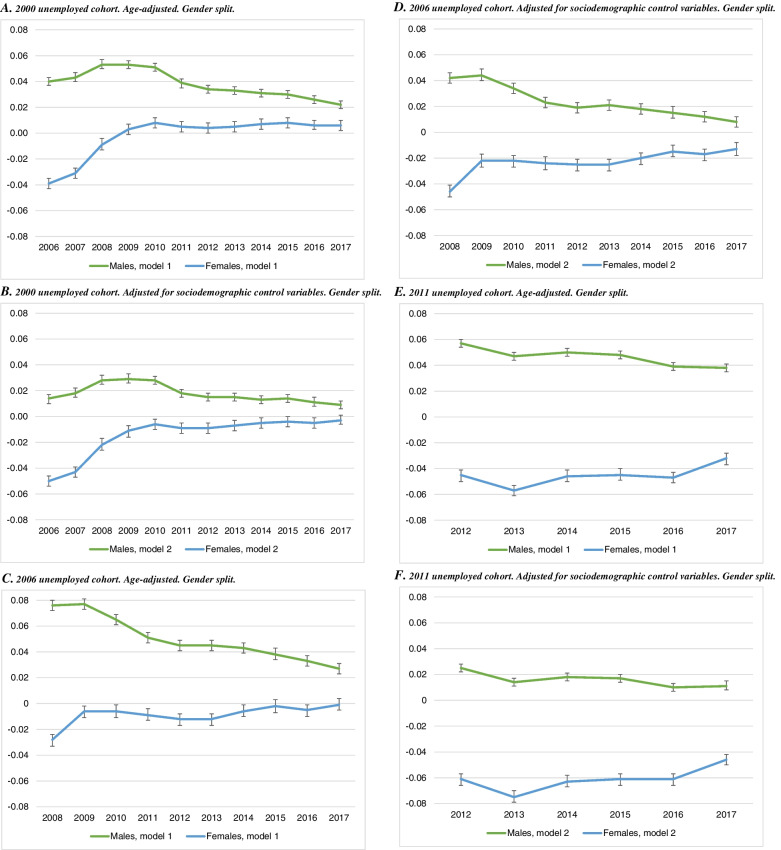
Linear probability models of receiving sick pay in 2006–2017, by unemployment

The results for receiving sick pay are presented in Fig. 2. Unemployed males in the 2000 cohort have a higher likelihood, between 2 and 5 percentage points, of receiving sick pay in the years following unemployment during 2006–2017 (panel A). Unemployed females, by comparison, have a lower or similar probability of receiving sick pay compared with the employed. The gender differences are noticeable also after adjustment for sociodemographic covariates (panel B). Similar gender differences appear in the 2006 (panels C and D) and 2011 cohorts (panels E and F) as well.

**Fig. 3:**
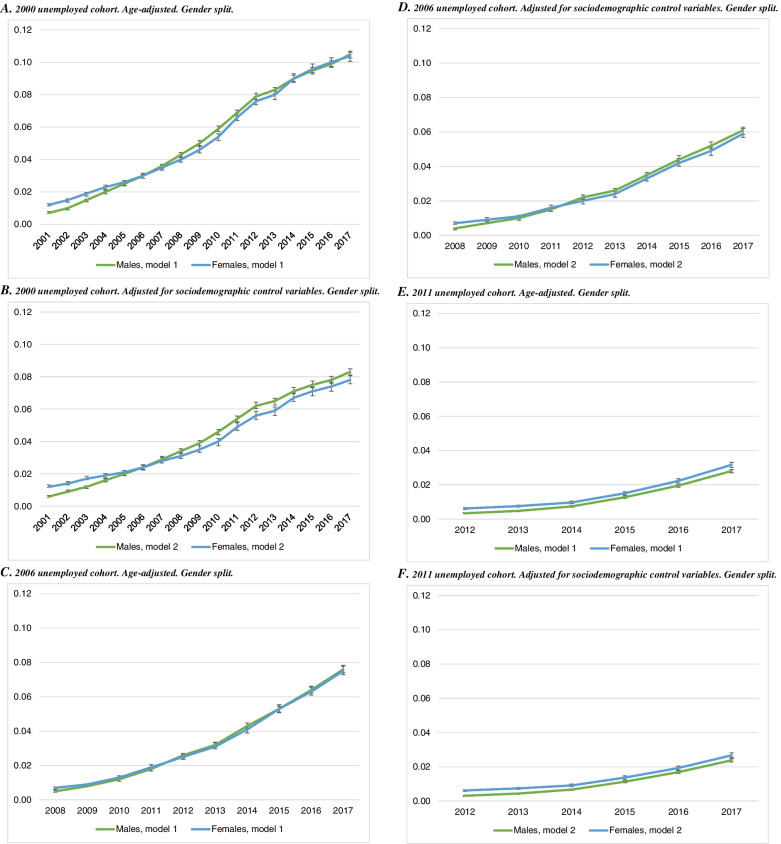
Linear probability model of disability benefit utilisation in 2001–2017, by unemployment

Figure 3 presents the findings for disability benefit utilisation. The empirical findings are practically identical for men and women for the 2000 cohort, with a small coefficient initially that grows slowly over time (panel A). Towards the end of the observation period, unemployed men and women are roughly 8 percentage points more likely than the employed to receive disability benefits (panel B). Both the increasing coefficient size over time and the lack of gender differences appear in the 2006 (panels C and D) and 2011 cohorts (panels E and F) as well.

**Fig. 4:**
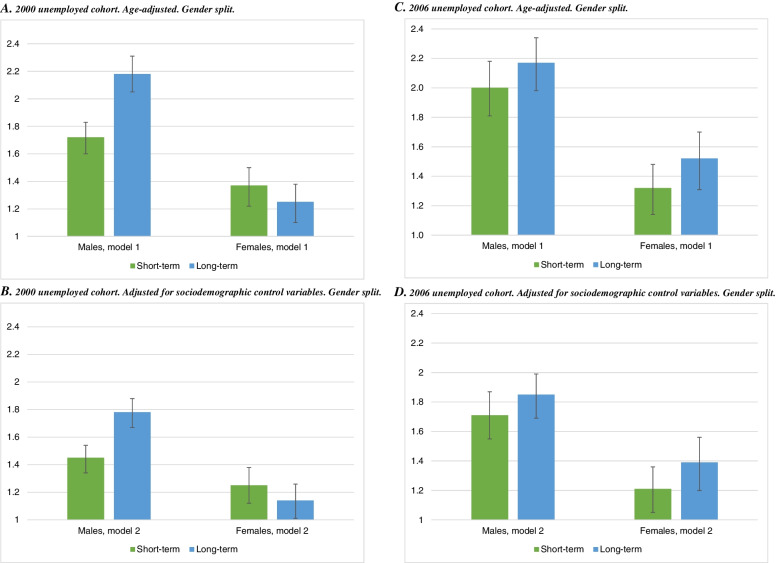
Logistic regression of 10-year mortality likelihood, by short- and long-term unemployment

Finally, the results for 10-year mortality likelihood are presented in Fig. 4, with odds ratios derived from logistic regression. A proxy measure of unemployment length is included in the models by differentiating between those who received less (i.e., short-term) or more (i.e., long-term) than the median amount of unemployment benefits during 2000/2006. The odds ratio is considerably larger for males than females, for both short- and long-term unemployment in 2000 (panels A and B). The excess mortality is lower, but still noticeable in model 2 (1.45; 1.78, panel B) than in the age-adjusted model (1.72; 2.18, panel A) for males. Adjustment for sociodemographic covariates attenuates the odds ratio less for females. The empirical pattern is very similar for the 2006 cohort (panels C and D), although the odds ratios are somewhat larger. Similar findings appear for 6-year mortality likelihood, where all three unemployed cohorts can be included (Figure A1, additional file 1). The results derived from the linear probability models, that are easier to compare between different samples and model specifications, confirm the presented mortality findings (Figure A2, additional file 2).

### Robustness checks

The results were similar when a broader definition of ‘employed’ (i.e., income from work above zero in 2000/20006/2011) was used as a control group. A similar empirical pattern appeared when short-term unemployment (i.e., those who received less than the median amount of unemployment benefit) were excluded from the regressions. This result confirms a recent meta-analysis [51], which found that it is unemployment occurrence that matters for health, rather than the length of the unemployment spell.

By re-running the analyses for the 2011 unemployed cohort on observational years preceding the unemployment experience (i.e., until 2010), we can gain some insights into the importance of health-related social mobility. Hospitalisation was somewhat higher among the 2011 unemployed cohort even before the unemployment experience (i.e., during 2008–2010). However, the coefficients are noticeably smaller for both males (0.026–0.031, model 1) and females (0.017–0.019, model 1), compared to the results presented in Fig. 1. Receiving sick pay was elevated – roughly 10 and 12 percentage points for males and females, respectively (model 1) – in 2009 and 2010 for those who experienced unemployment in 2011. Disability benefit utilisation was not higher, however, during 2001–2010 among those who became unemployed in 2011, compared to people with firm employment. Thus, health-related social mobility plays a role, but can only account for a portion of the health consequences demonstrated in this paper. Furthermore, the empirical findings for the 2011 unemployed cohort remain robust after adjusting for previous health problems (i.e., any sick pay receipt or hospitalisation for the five above-mentioned ICD-code groups during 2008–2010) in the regression models (see Figures A3–A6, additional files 3, 4, 5 and 6).

Educational inequalities for the 2011 unemployed cohort were examined (results from model 1 reported in parentheses). The differences between the low and high educated is small for hospitalisation, among both males (0.025–0.037 vs. 0.030–0.042) and females (0.014–0.027 vs. 0.020–0.035). Sick pay receipt is elevated mainly among unemployed males with high education (0.034–0.056 vs. -0.011–0.013). Sick pay receipt is especially low, roughly minus 7 to 10 percentage points, among unemployed females with low education, whereas high-educated unemployed females differ less (roughly minus 2 to 6 percentage points). Disability benefit utilisation is higher among unemployed males (0.006–0.038 vs. 0.002–0.019) and females (0.011–0.044 vs. 0.003–0.017) with low education, compared to the unemployed with high education. Excess mortality is similar for low and high educated, among both men (OR = 1.49 vs. 1.42) and women (OR = 1.27 vs. 1.22).

It is not random who becomes unemployed; previous employment history can, for instance, play an important role. This question was examined by including indicators for unstable employment records during 2000–2009 in regressions with unemployment in 2011 as the outcome (see Figures A7 and A8, additional files 7 and 8). The empirical results indicate that previous receipt of unemployment benefits, and especially during the later period (i.e., 2007–2009), is a strong predictor of unemployment in 2011. However, weak labour market attachment, operationalized as earning less than 1 BA in work income, appears to be less decisive. The gender differences are small overall for both indicators.

## Discussion

The present paper analysed Norwegian administrative register data and examined the following overarching research question: How gendered are the health consequences of unemployment in Norway from 2000 to 2017? The empirical findings reveal that the gender differences in health consequences of unemployment are quite significant overall in the gender-egalitarian Norwegian society. More specifically, males are affected more by unemployment than females, as evidenced by:

(i) somewhat higher hospitalisation,
(ii) considerably higher sick pay receipt, and
(iii) more noticeable excess mortality a decade after the unemployment experience.

There is no gender component at all, however, in disability benefit utilisation, where the results are practically identical for men and women. The gender differences were quite small for hospitalisation and relatively large for mortality – a finding that is in accordance with the ‘gender health paradox’ and therefore lends some support to hypothesis 1. The health consequences appear to be very stable over time, as the results are similar for three different unemployment cohorts (2000; 2006; 2011). The gender differences have not decreased noticeably over the observation period, and there is thus little or no empirical support for hypothesis 2.

According to the World Economic Forum’s Global Gender Gap Report 2021, Norway is the third most gender equal country in the world, after Iceland and Finland [42]. Only Iceland and Sweden out of 34 European countries had higher female labour force participation (age 25–64) than Norway in 2017 [43]. The health consequences of unemployment nonetheless appear to be quite gendered in the gender-egalitarian Norwegian context, a finding that does not align particularly well with the social role theory. Yet, cross-national comparative research – with sufficient variation across countries and regions in gender equality – is needed to properly test the explanatory power of the social role theory.

It is important to note that women also experience negative health consequences due to unemployment (e.g., higher hospitalisation rates and disability benefit utilisation), yet not as often as men do. Consequently, the question is not whether men and women are harmed by unemployment, but rather why there is an overrepresentation of undesirable health consequences among unemployed men. Gender segregation in the labour market, that is, the types of jobs and working conditions that men and women typically hold (and lose), may be one potential explanation, although it seems unlikely, as the Norwegian labour market has become more gender equal since the turn of the century [52].

There was a striking resemblance in the empirical results for three different unemployed cohorts (2000; 2006; 2011). The strategy of choosing exposure years with as similar economic conditions as possible should, in theory, minimise cross-cohort variation (e.g., compositional differences in unobservable characteristics). Thus, this comparative design sacrifices some variation to isolate the time trend. There are drawbacks to this design and uncertainties remain. Nonetheless, the findings clearly demonstrate that people who were unemployed in comparable economic conditions experienced very similar consequences in the subsequent years, despite noticeable demographic changes during 2000–2011, that is, more people attaining long education, fewer people getting married, and an increasing share with immigrant background (cf., Table 1). Stability over time in the health consequences of unemployment indicates that real policy change is needed to ameliorate the situation to any meaningful extent. The minor adjustments of the unemployment insurance system in Norway since the turn of the century have largely failed to keep unemployed men and women healthy.

Future research should dig deeper into the gendered health consequences of unemployment, preferably by linking register data with longitudinal survey data, including detailed information on the social surroundings and life circumstances of individuals in, outside, and on the fringes of the labour market. Theoretically driven empirical research that, for example, compares the explanatory power of the social role theory to other theoretical models is especially needed.

### Strengths and weaknesses

The current study used high-quality population-wide administrative register data sources with minimal measurement error and no attrition. Unemployment benefit recipients were followed longitudinally over the period 2000 to 2017, and we can therefore gain some insight into the medium- and long-term health consequences. Results for four different health outcomes (hospitalisation, sick pay receipt, disability benefit utilisation, and mortality) were presented, thus painting a comprehensive picture of the gendered health consequences. Time trends were assessed by analysing three unemployed cohorts (2000; 2006; 2011) that experienced unemployment during similar economic conditions.

The external validity of this paper may be limited due to the booming economic conditions in Norway during 2000–2017 (Figure A9, additional file 9), but the empirical results can still be of some relevance for other European countries. Employment, or lack thereof, is an important social determinant of health. It therefore seems likely that the public health impact of unemployment is greater in countries with poorer economic conditions and less developed social welfare systems. Furthermore, men experience more noticeable negative health consequences due to unemployment than women do, even in a gender-egalitarian country such as Norway, perhaps because men to a larger extent ‘suffer in silence’ during and after unemployment. The health consequences of unemployment could be even more gendered in countries where the male breadwinner model still prevails.

The assumption that the three unemployed cohorts have a similar composition, and can thus be compared, might not hold. Even though the economic conditions were similar in 2000, 2006, and 2011, as evidenced by unemployment rates [47, 48], there could still be cohort differences in unobserved characteristics of relevance for health and mortality. Furthermore, the economic conditions in the years following the unemployment experience varied somewhat for the three cohorts (Figure A9, additional file 9), perhaps implying cohort differences in, for example, re-employment likelihood. A 2003 policy change reduced the maximum entitlement period for unemployment benefits from three to two years, which might have affected the results (e.g., more stress and financial strain for the two most recent cohorts).

Most findings in this study were derived from OLS regressions, and there is a risk that the underlying assumptions do not hold. Hellevik has demonstrated that violation of the homoscedasticity assumption has little practical importance for significance testing [53]. It is also reassuring that the mortality results were qualitatively similar when running both linear and logistic models. The analyses of hospitalisation, sick pay receipt, and disability benefit utilisation were estimated with logistic regression as well (see Figures A10–A12, additional files 10, 11 and 12). Overall, the empirical results derived from logistic regression confirm the findings from the linear models, with one exception: there are somewhat more gender differences in the odds ratios for disability benefit utilisation post-unemployment.

Certain characteristics of importance for the health consequences of unemployment were omitted from this study. One example is parental background. Poverty, labour market disadvantages, and other unfavourable social circumstances may be transmitted across generations. Gender differences, and potential changes over time, in intergenerational transmission of various social and economic disadvantages may be an interesting avenue for future research.

The current study established statistical associations by describing various health consequences post-unemployment. However, the empirical findings cannot be interpreted as causal within a counterfactual framework. Propensity score matching could be one way to come closer to establishing causal effects for future research on the current topic.

Finally, many people who are unemployed in Norway, roughly half [54], are not covered by the Norwegian unemployment insurance system. People with low total work income (i.e., below 1,5 BA), typically due to a weak labour market attachment and vulnerable life circumstances are, for example, not entitled to unemployment benefits. Thus, the empirical results presented here are most likely downwardly biased, in other words, the negative health consequences would be greater if more unemployed people in Norway were eligible for unemployment benefits and thus included in the current study.

## Conclusion

This paper illuminated the gendered health consequences of unemployment using Norwegian register data covering the years 2000 to 2017. The empirical findings reveal a strong gender component, with unemployed males being slightly more prone to hospitalisation, and considerably more likely to be the recipients of sick pay, than unemployed females. Men have an elevated mortality risk a decade after experiencing unemployment, whereas excess mortality is less noticeable among unemployed women. There is no gender component whatsoever though in disability benefit utilisation post-unemployment. Comparing the findings for three different unemployed cohorts (2000, 2006, and 2011), we found a remarkable pattern of stability over time. Overall, the current study demonstrates that the health consequences of unemployment are serious, gendered, and enduring in Norway.

## Supplementary Information

**Additional file 1:** **Figure A1.** Logistic regression of 6-year mortality likelihood, by short- and long-term unemployment.

**Additional file 2:** **Figure A2.** Linear regression of 10-year mortality likelihood, by short- and long-term unemployment.

**Additional file 3:** **Figure A3.** Linear probability models of hospitalisation 2012-2017, by unemployment.

**Additional file 4:** **Figure A4.** Linear probability models of sick pay receipt 2012-2017, by unemployment.

**Additional file 5:** **Figure A5.** Linear probability models of disability benefit utilisation 2012-2017, by unemployment.

**Additional file 6:** **Figure A6.** Logistic regression of 6-year mortality likelihood, by short- and long-term unemployment.

**Additional file 7:** **Figure A7.** Linear probability models of unemployment 2011, by unemployment 2000-2009.

**Additional file 8:** **Figure A8.** Linear probability models of unemployment 2011, by weak labour market attachment 2000-2009 (i.e., earned less than 1 BA in work income).

**Additional file 9:** **Figure A9.** Unemployment rate 2000-2017, yearly average (source: NAV 2021a).

**Additional file 10:** **Figure A10.** Logistic regression of hospitalisation 2008-2017, by unemployment.

**Additional file 11:** **Figure A11.** Logistic regression of sick pay receipt 2006-2017, by unemployment.

**Additional file 12:** **Figure A12.** Logistic regression of disability benefit utilisation 2001-2017, by unemployment.

## Data Availability

The administrative register data that support the findings of this study cannot be publicly shared due to Norwegian data protection laws. However, the register data can be made available by the data owners (i.e., Statistics Norway and the Norwegian Directorate of Health). Researchers can apply for access to the information that is available in the public administrative registries in Norway. If the application is granted, researchers will gain access to the data in anonymised and deidentified format, but only on a temporary basis (e.g., during the lifespan of a research project).

## Abbreviations

BA: Base amount
ICD: International Classification of Diseases
ISCED: International Standard Classification of Education
NOK: Norwegian krone
NPR: Norwegian Patient Registry
OLS: Ordinary least squares
OR: Odds ratio
