# Supplementary material for: Gendered health consequences of unemployment in Norway 2000–2017: a register-based study of hospital admissions, health-related benefit utilisation, and mortality
Source: BMC Public Health. 2022 Dec 28;22:2447. doi: 10.1186/s12889-022-14899-8 (PMC9795737; doi:10.1186/s12889-022-14899-8)

Additional file 2

Figure A2. Linear regression of 10-year mortality likelihood, by short- and long-term unemployment.

Panel A. 2000 unemployed cohort. Age-adjusted. Gender split.

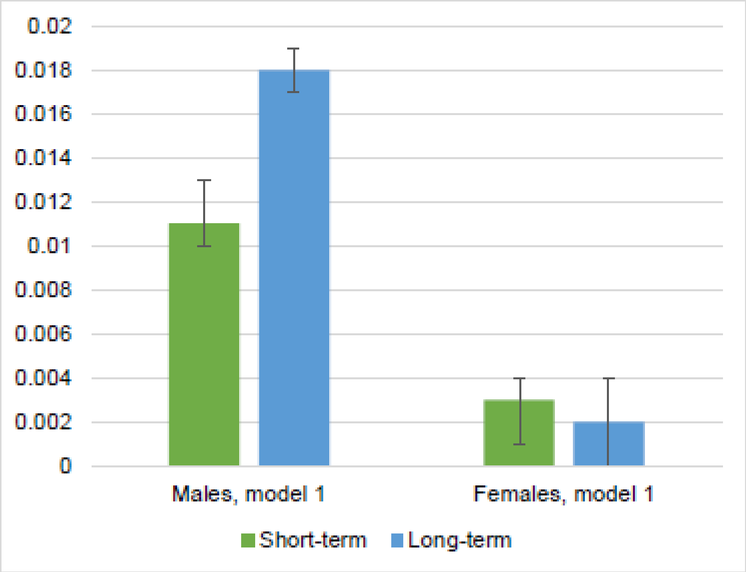

Panel B. 2000 unemployed cohort. Adjusted for sociodemographic control variables. Gender split.

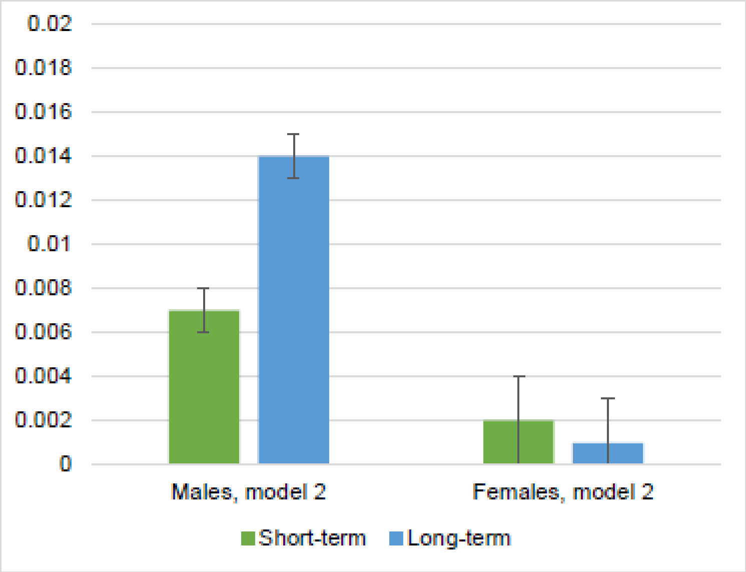

Panel C. 2006 unemployed cohort. Age-adjusted. Gender split.

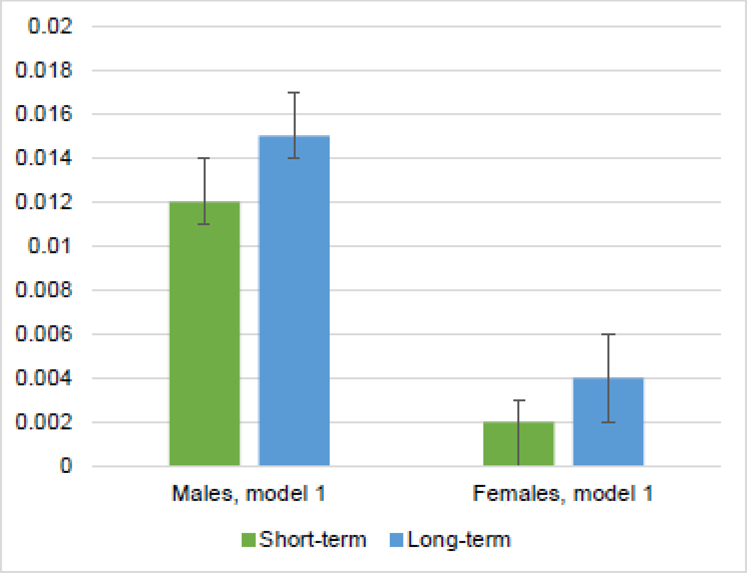

Panel D. 2006 unemployed cohort. Adjusted for sociodemographic control variables. Gender split.

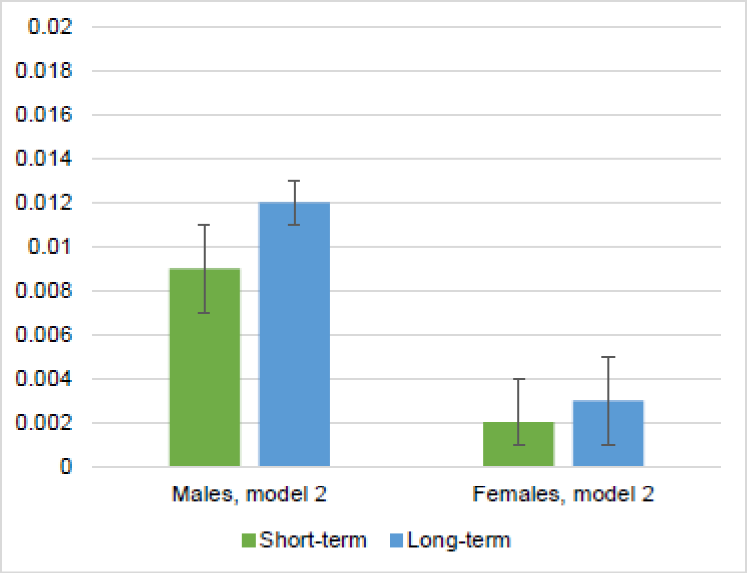

Supplement: Supplementary file 2 — Additional file 2: Figure A2. Linear regression of 10-year mortality likelihood, by short- and long-term unemployment. [file 12889_2022_14899_MOESM2_ESM.pdf]
