# Supplementary material for: Gendered health consequences of unemployment in Norway 2000–2017: a register-based study of hospital admissions, health-related benefit utilisation, and mortality
Source: BMC Public Health. 2022 Dec 28;22:2447. doi: 10.1186/s12889-022-14899-8 (PMC9795737; doi:10.1186/s12889-022-14899-8)

Additional file 3

Figure A3. Linear probability models of hospitalisation 2012-2017, by unemployment.

Panel A. 2011 unemployed cohort. Adjusted for age and sick pay receipt 2008-2010. Gender split.

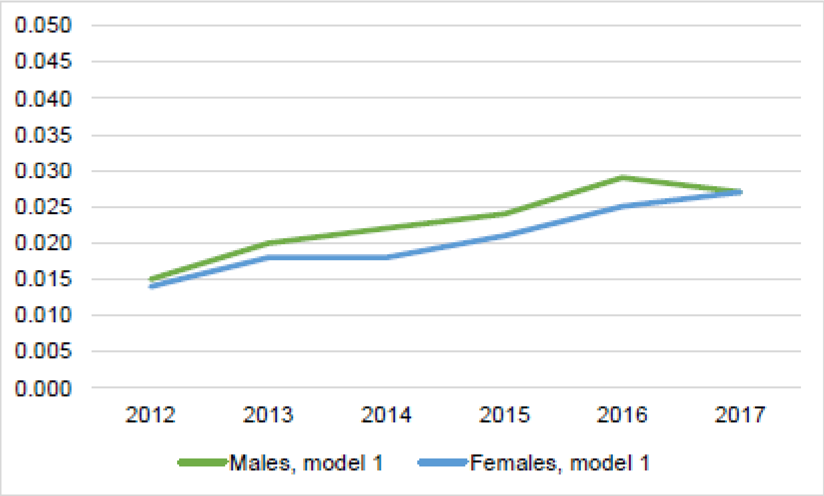

Panel B. 2011 unemployed cohort. Adjusted for age, sick pay receipt 2008-2010, and sociodemographic control variables. Gender split.

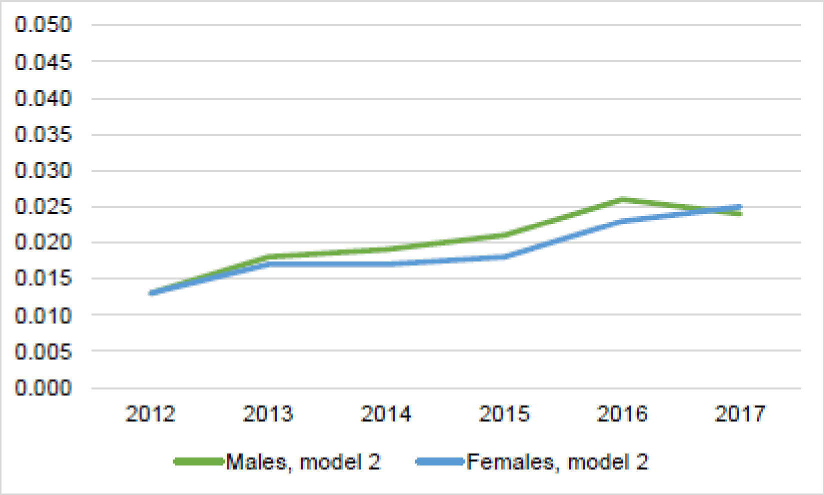

Supplement: Supplementary file 3 — Additional file 3: Figure A3. Linear probability models of hospitalisation 2012-2017, by unemployment. [file 12889_2022_14899_MOESM3_ESM.pdf]
