# Supplementary material for: Gendered health consequences of unemployment in Norway 2000–2017: a register-based study of hospital admissions, health-related benefit utilisation, and mortality
Source: BMC Public Health. 2022 Dec 28;22:2447. doi: 10.1186/s12889-022-14899-8 (PMC9795737; doi:10.1186/s12889-022-14899-8)

Additional file 7

Figure A7. Linear probability models of unemployment 2011, by unemployment 2000-2009.

Panel A. Age-adjusted. Gender split.

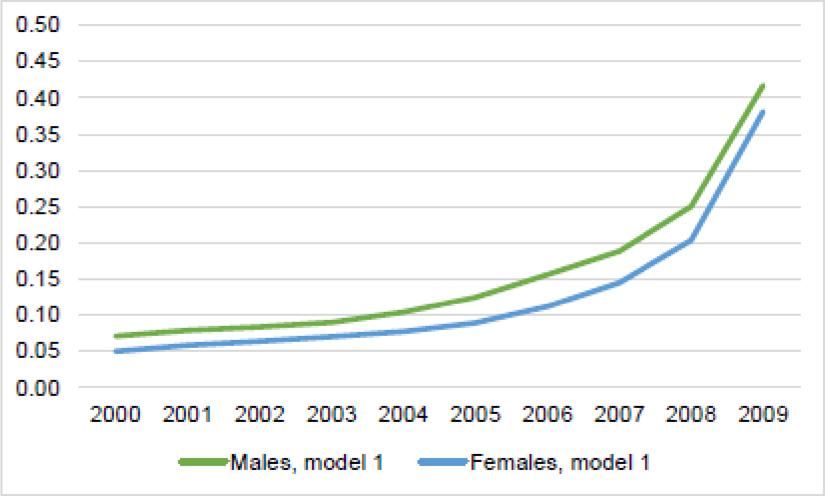

Panel B. Adjusted for sociodemographic control variables. Gender split.

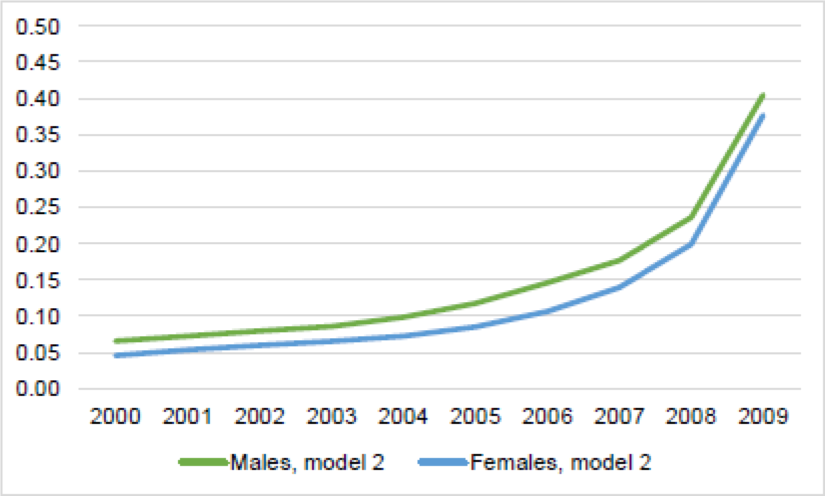

Supplement: Supplementary file 7 — Additional file 7: Figure A7. Linear probability models of unemployment 2011, by unemployment 2000-2009. [file 12889_2022_14899_MOESM7_ESM.pdf]
