# Supplementary material for: Gendered health consequences of unemployment in Norway 2000–2017: a register-based study of hospital admissions, health-related benefit utilisation, and mortality
Source: BMC Public Health. 2022 Dec 28;22:2447. doi: 10.1186/s12889-022-14899-8 (PMC9795737; doi:10.1186/s12889-022-14899-8)

Additional file 8

Figure A8. Linear probability models of unemployment 2011, by weak labour market attachment 2000-2009 (i.e., earned less than 1 BA in work income).

Panel A. Age-adjusted. Gender split.

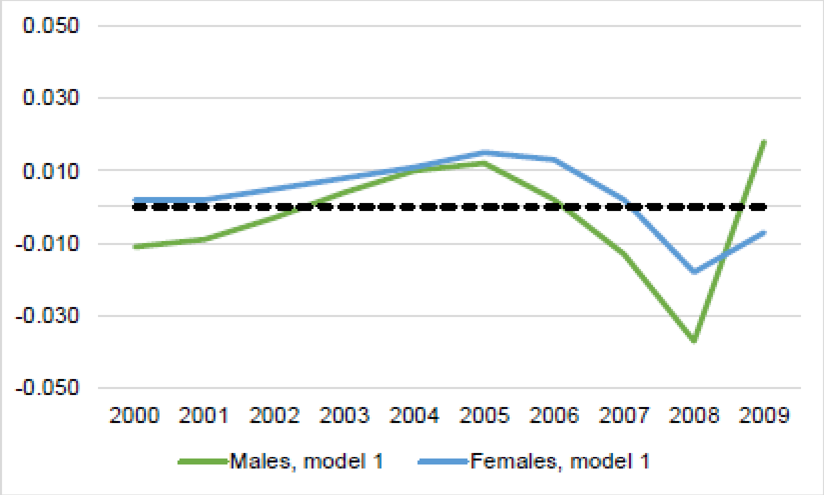

Panel B. Adjusted for sociodemographic control variables. Gender split.

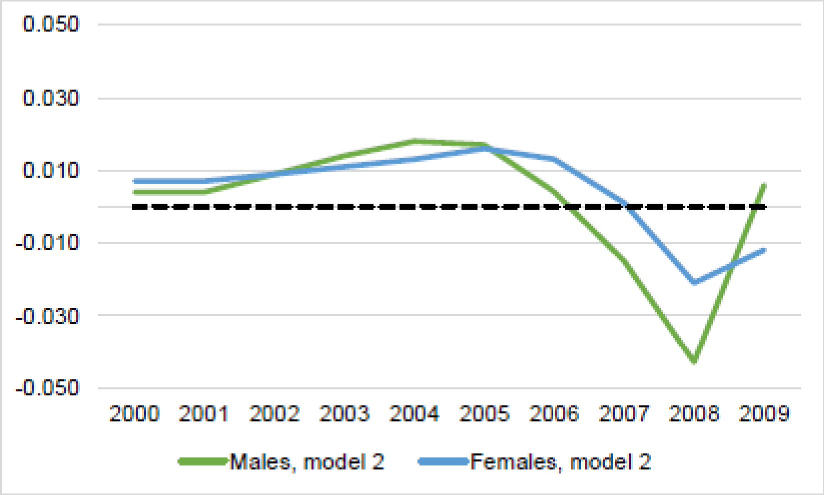

Supplement: Supplementary file 8 — Additional file 8: Figure A8. Linear probability models of unemployment 2011, by weak labour market attachment 2000-2009 (i.e., earned less than 1 BA in work income). [file 12889_2022_14899_MOESM8_ESM.pdf]
