# Supplementary figures and images for: Gendered health consequences of unemployment in Norway 2000–2017: a register-based study of hospital admissions, health-related benefit utilisation, and mortality
Source: BMC Public Health. 2022 Dec 28;22:2447. doi: 10.1186/s12889-022-14899-8 (PMC9795737; doi:10.1186/s12889-022-14899-8)

**Additional file 9**

*Figure A9. Unemployment rate 2000-2017, yearly average (source: NAV 2021a).*

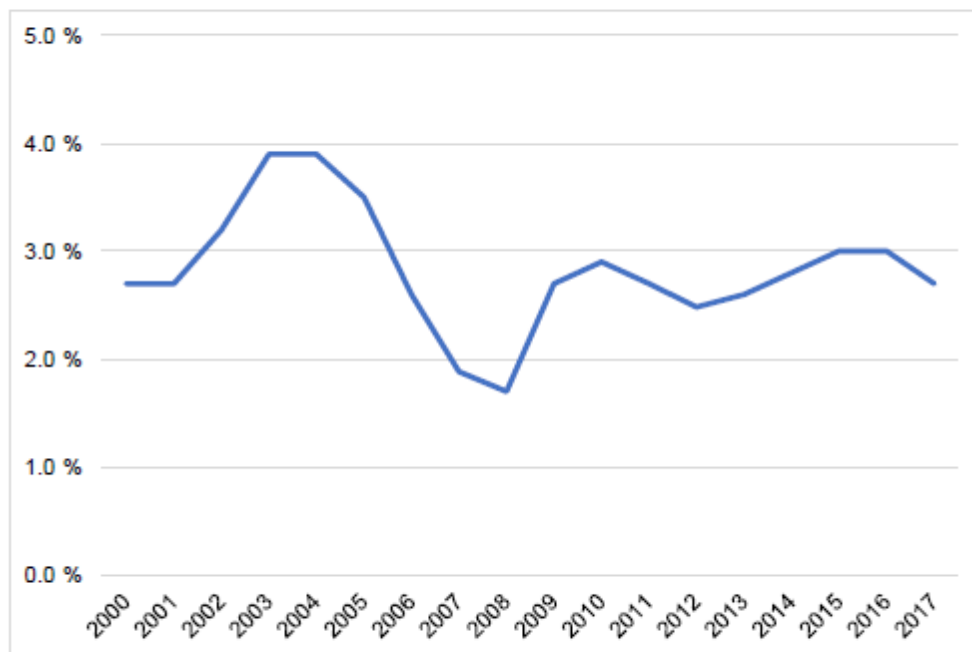

Supplement: Supplementary file 9 — Additional file 9: Figure A9. Unemployment rate 2000-2017, yearly average (source: NAV 2021a). [file 12889_2022_14899_MOESM9_ESM.pdf]
